# LIFE-COURSE MULTI-DISCIPLINARY PREDICTORS OF DEMENTIA AMONG OLDER ADULTS

**DOI:** 10.1093/geroni/igac059.2802

**Published:** 2022-12-20

**Authors:** Sayaka Kuwayama, Kevin González, Freddie Márquez, Hector González, Wassim Tarraf

**Affiliations:** University of California San Diego, La Jolla, California, United States; University of California San Diego, La Jolla, California, United States; University of California San Diego, La Jolla, California, United States; University of California San Diego, La Jolla, California, United States; Wayne State University, Detroit, Michigan, United States

## Abstract

The incidence of dementia is rapidly increasing. Identifying risk factors for dementia may help improve risk assessment, increase awareness for risk reduction, and identify potential targets for interventions. We use a life-course multi-disciplinary modeling framework to examine leading predictors of incident dementia (ID). We use the Health and Retirement Study (HRS) to measure 57 exposures across 7 different domains: (1) demographic, (2) adverse childhood socioeconomic and psychosocial, (3) adverse adulthood experiences, (4) adult socioeconomic status, (5) health behaviors, (6) social connections, and (7) adult psychological conditions. Our outcome is ID (over 8-years) operationalized using Langa-Weir classification for adults aged 65+ years who meet criteria for cognitively normal at the baseline when all exposures are measured (Nf 1,622 in testing set and Nf1,460 in validation set). We compare standard methods (Logistic regression) with machine learning (ML) approaches (Lasso, Random Forest) in identifying highly predictive exposures across the risk domains of interest. Standard methods identified lower education, childhood financial duress, and pessimism as among the leading factors associated with ID. Psychological factors explained the highest variance for ID, followed by adult socioeconomic and adverse childhood factors. However, ML techniques differed in their identification of (1) predictors and (2) factors predictive importance. The findings emphasize the importance of upstream risk factors and the long-reach of childhood experiences on cognitive health. The ML approaches highlight the importance of life-course multi-disciplinary frameworks for improving dementia risk assessment. Further investigations are needed to identify how complex interactions of life-course risk factors can be addressed through interventions.

